# Functionalized Protein Nanotubes Based on the Bacteriophage vB_KleM-RaK2 Tail Sheath Protein

**DOI:** 10.3390/nano11113031

**Published:** 2021-11-12

**Authors:** Greta Labutytė, Simona Povilonienė, Eugenijus Šimoliūnas, Dovydas Gabrielaitis, Martynas Skapas, Algirdas Noreika, Rolandas Meškys, Vida Časaitė

**Affiliations:** 1 Department of Molecular Microbiology and Biotechnology, Institute of Biochemistry, Life Sciences Center, Vilnius University, Sauletekio av. 7, LT-10257 Vilnius, Lithuania; greta.labutyte@gmc.stud.vu.lt (G.L.); simona.poviloniene@bchi.vu.lt (S.P.); eugenijus.simoliunas@bchi.vu.lt (E.Š.); dovydas.gabrielaitis@gmc.vu.lt (D.G.); algirdas.noreika@gmc.vu.lt (A.N.); 2 Department of Neurobiology and Biophysics, Institute of Biosciences, Life Sciences Center, Vilnius University, Sauletekio av. 7, LT-10257 Vilnius, Lithuania; 3 Center for Physical Sciences and Technology, Saulėtekio av. 3, LT-10257 Vilnius, Lithuania; martynas.skapas@ftmc.lt

**Keywords:** bacteriophage vB_KleM-RaK2, tail sheath protein, nanotube, green fluorescent protein, mCherry, YqfB, self-assembly, fluorescent nanoparticles, functionalized nanoparticle

## Abstract

We report on the construction of functionalized nanotubes based on tail sheath protein 041 from vB_KleM-RaK2 bacteriophage. The truncated 041 protein (041Δ200) was fused with fluorescent proteins GFP and mCherry or amidohydrolase YqfB. The generated chimeric proteins were successfully synthesized in *E. coli* BL21 (DE3) cells and self-assembled into tubular structures. We detected the fluorescence of the structures, which was confirmed by stimulated emission depletion microscopy. When 041Δ200GFP and 041Δ200mCherry were coexpressed in *E. coli* BL21 (DE3) cells, the formed nanotubes generated Förster resonance energy transfer, indicating that both fluorescent proteins assemble into a single nanotube. Chimeric 041Δ200YqfB nanotubes possessed an enzymatic activity, which was confirmed by hydrolysis of *N*^4^-acetyl-2′-deoxycytidine. The enzymatic properties of 041Δ200YqfB were similar to those of a free wild-type YqfB. Hence, we conclude that 041-based chimeric nanotubes have the potential for the development of delivery vehicles and targeted imaging and are applicable as scaffolds for biocatalysts.

## 1. Introduction

Nanoscale materials based on self-assembly of proteins are used for various purposes, including the formation of structurally different shapes; the development of biosensors; the manufacture of optical, conductive, semiconductor, and magnetic nanoelectronics materials; as well as in gene and drug-delivery devices and vaccines [1,2,3]. Viruses, as a natural source of countless self-assembling proteins, are particularly widely studied and adapted for these purposes [4,5]. They come in a wide variety of shapes and sizes. Due to their great diversity, viruses can be applied in many areas. New bioconjugation and chemical-modification methods have evolved to provide functional modification, both externally and internally, on virus protein scaffolds. For instance, a chemical-modification strategy has been used for the immobilization of enzymes through streptavidin-biotin assembly or glutaraldehyde [6,7]. However, these methods have some disadvantages, such as multistep and low efficiency. On the other hand, the fusion strategy, successfully used for introducing some peptides [8,9,10,11,12] or functionally active proteins [13,14,15], is characterized by simplicity and productivity of the synthesis [8,9,10,11,12,13,14,15]. However, only a few structures are created by gene fusion, as the attached protein can disturb the assembly or may lose its functional activity [16]. Hence, the development and testing of novel nanomaterials are crucial for further progress in this area.

The tails of *Myoviridae* bacteriophage are complex structures consisting of a baseplate with tail fibers and a long, non-contractible tube surrounded by a contractile sheath. The tail is employed for recognition and attachment to the host cell. Easy synthesis and assembly, as well as some chemical properties, make phage tail self-assembling proteins attractive for functional modifications [16,17,18,19]. Tail sheath proteins can self-assemble both in vivo and in vitro into tubular structures of variable lengths, called polysheaths [20]. The large and wide inner diameter of the tail sheath can be expected to be a promising scaffold for the generation of functional nanostructures. Some attempts were made to explore these structures for functional modifications [17]; however, fusion with active proteins has not been achieved.

Here, we present results of characterization and application of tail sheath protein 041 of bacteriophage vB_KleM-RaK2 [21,22]. This giant virus of the family *Myoviridae* infects *Klebsiella* sp. bacteria and has a particularly large genome (345,809 bp). Bacteriophage vB_KleM-RaK2 is characterized by an isometric head of 123 nm in diameter and a contractile tail of 128 nm in length and 21.5 nm or 42 nm in width in its extended or contracted state, respectively. We showed that functionally modified nanotubes could be generated using 041 protein fused with fluorescent proteins or YqfB-type amidohydrolase.

## 2. Materials and Methods

### 2.1. Materials

Restriction endonucleases (HindIII, XhoI, NcoI, SnaBI, NheI), T4 DNA ligase, Phusion High-Fidelity PCR Master Mix, PageRulerTM Prestained Protein Ladder, and Pierce™ Coomassie Plus Assay Reagent were purchased from Thermo Fisher Scientific, Vilnius, Lithuania. pET21d and pET28b vectors were purchased from Novagen, Madison, WI, USA. Nutrient medium was purchased from Roth, Karlsruhe, Germany. Sucrose and salts were purchased from Sigma-Aldrich, Buchs, Switzerland. TSKgel G4000SWXL was obtained from Tosoh Bioscience, Tokyo, Japan. *E. coli* DH5α (Thermo Fisher Scientific, Vilnius, Lithuania) and BL21 (DE3) (Novagene, Madison, WI, USA) were used for cloning and expression experiments.

### 2.2. Gene Construction

The sequence of ORF041 of vB_KleM-RaK2 was amplified with Phusion DNA High-Fidelity PCR Master Mix using the bacteriophage genomic DNA [22] and primers 041NcoF and 041XhoR (Table 1). The PCR product was digested with NcoI and XhoI and cloned into the corresponding restriction sites of plasmid pET21d (ampicillin resistant). The resulting plasmid was named pET21_041. Then, 300, 450, and 600 bp-truncated 041 genes (deletions of the C-end of the encoded protein) were amplified with the forward primer 041NheF and 041Δ100HindR, or 041Δ150HindR, or 041Δ200HindR (Supplementary Table S1) as a reverse primer, respectively. The PCR products were digested with NheI and HindIII and cloned into the corresponding restrictions sites of plasmid pET28b (kanamycin resistant). The 041Δ200 fragment was also cloned into the NcoI and XhoI-digested pET21d. The GFP (GFPmut2) [23] and mCherry [24] genes were PCR amplified using primer pairs gfpHindF/gfpXhoR and mcheryHindF/mcheryXhoR, respectively (Table 1), digested with HindIII and XhoI, and cloned into the corresponding restrictions sites of plasmid pET28_041Δ200 (GFP) or pET21_041Δ200 (mCherry). The YqfB gene was fused to fragment 041Δ200 with the GS linker in between. The GS linker was constructed from two single-stranded DNA fragments, GS_F and GS_R, hybridized to each other. The linker was cloned into plasmid pET21_041Δ200 via XhoI and HindIII cleavage sites. YqfB was amplified with the primers YqfbbukF and YqfbbukR and ligated into the plasmid pET28_041Δ200_GS via the SnaBI cleavage site. The *E. coli* DH5α cells transformed with recombinant plasmids were cultivated on LB agar supplemented with 50 μg/mL ampicillin or 40 μg/mL kanamycin.

### 2.3. Expression and Purification of 041 and Chimeras

*E. coli* BL21 (DE3) cells transformed with recombinant plasmids were grown in 50 mL LB medium containing ampicillin or kanamycin at 30 °C with aeration. Protein expression was induced by adding 0.1 mM IPTG at 0.6–0.8 OD_600_, and cells were grown for a further 18 h at 20 °C. Cell biomass was suspended in 5 mL of buffer A (50 mM Tris-HCl, 0.01% TritonX-100, pH 8) and disrupted by sonication at 30% amplitude for 2 min (1 s on/3 s off) by using the Branson Ultrasonics™ Sonifier™ SFX250 (St. Louis, MO, USA). A lysate was cleared by centrifugation at 10,000× *g* for 20 min. and was applied onto 10 mL 40% and 10 mL 30% (*w*/*v*) sucrose gradient in buffer A. The proteins were sedimented by centrifugation at 29,000× *g* for 2 h at 4 °C (Beckman Coulter Optima L-90 K ultracentrifuge, rotor 60Ti, Indianapolis, IN, USA). The protein pellets were resuspended in buffer A.

A high-performance liquid chromatograph (HPLC) with UV–vis and fluorescence detection system (Shimadzu, Kyoto, Japan) was used for analysis on TSKgel G4000SWXL column, equilibrated in 10 mM Tris-HCl, pH 8.0. The proteins were analyzed by SDS-PAGE (14% separating and 4.0% stacking gels) according to Laemmli [25]. The concentration of protein was determined using Pierce™ Coomassie Plus (Bradford) Assay Reagent by standard microplate protocol.

### 2.4. Transmission Electron Microscopy (TEM)

The images of 041 were obtained by transmission electron microscopy of the negatively-stained samples, as described in [26]. A total of 10 µL (0.2 mg/mL) of the sample solution was directly applied on the carbon-coated nickel grid (Agar Scientific, Essex, UK), and the excess liquid was drained with filter paper before staining with two successive drops of 2% uranyl acetate (pH 4.5). The prepared sample was dried and examined with a Morgagni 268(D) transmission electron microscope (FEI, Hillsboro, OR, USA).

### 2.5. Fluorescence Microscopy

Fluorescence activity and Förster resonance energy transfer (FRET) were confirmed by Leica TCS SP8 STED Nanoscope (Leica, Wetzlar, Germany). FRET efficiency was measured by fluorescence lifetime imaging microscopy (FLIM) and calculated using the Leica Application Suite X (Version: 3.5.6.21594). A 63 × oil (NA 1.4) objective and a white-light laser were used for excitation. Fluorescence emission spectra were recorded, as indicated in Table 2.

### 2.6. Amidohydrolase Activity Assay

Activity of amidohydrolase was determined spectrophotometrically using *N*^4^-acetyl-2′-deoxycytidine as the substrate. The reaction mixture (1 mL) consisted of 10 mM Tris-HCl buffer (pH 8.0), 0.2 mM of substrate, and 10 µL (0.2 mg/mL) of the enzyme solution. Hydrolysis of substrate was monitored by the decrease in absorbance at 310 nm (ε = 5361 M^−1^ cm^−1^) at 22 °C. One unit was defined as an amount of enzyme that hydrolyzed 1 µmol of *N*^4^-acetyl-2′-deoxycytidine per min.

The kinetic parameters Km and V_max_ for the enzyme were determined from the linear regression of Lineweaver–Burk double reciprocal plots obtained by varying the final concentration of *N*^4^-acetyl-2′-deoxycytidine in the reaction mixture from 0.05 to 0.5 mM.

The optimal pH of amidohydrolase was determined using 60 mM Britton-Robinson buffer in a pH range from 4.0 to 11.0. Enzyme stability at various pH levels was evaluated by pre-incubating the enzyme in respective buffers for 2 and 24 h. The optimal temperature was determined to be between 15 and 50 °C. The thermostability was assessed by pre-incubating the enzyme for 2 and 24 h at respective temperatures (30–60 °C). The relative activity was considered as 100% at optimal pH and temperature. To assess stability, the residual activity was considered as 100% without pre-incubation at respective pH and temperature. Activity values were calculated from at least three measurements.

### 2.7. Bioinformatics Analysis

The bioinformatic analysis of the 041 gene was performed using FASTA nucleotide, FASTA protein, and BLASTP. The phylogenetic analysis was conducted using MEGA 7 based on the alignment of the amino acid sequences of vB_KleM-RaK2 gp041 and its closest homologues in the reference proteins database. To find 041 protein structural homologs, the HHpred server was also used [27]. The 3D model of 041 protein was built using (PS)^2^: protein structure prediction server version 3.0 [28] and visualized using UCSF Chimera 1.13.1 software [29].

## 3. Results

### 3.1. Recombinant 041 Protein Analysis

**Figure 1:**
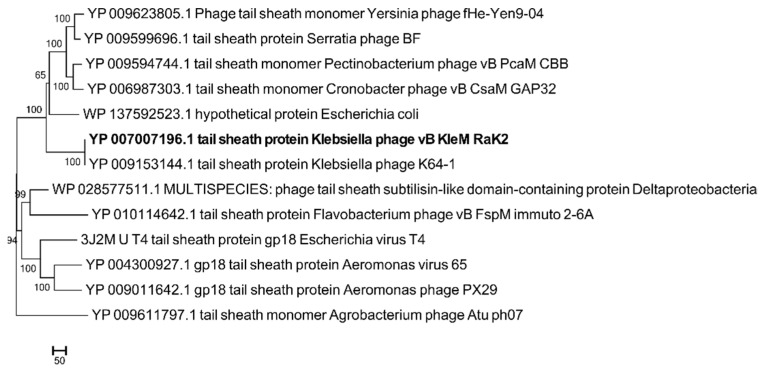
Phylogenetic analysis of protein 041 (**bold**) with selected structural proteins. The evolutionary history was inferred using the neighbor-joining method [31]. The percentage of replicate trees in which the associated taxa clustered together in the bootstrap test (2000 replicates) is shown next to the branches [32]. The evolutionary distances were computed using the number of differences method [33] and are in the units of the number of amino acid differences per sequence. Evolutionary analyses were conducted in MEGA7 [34].

Based on the results of BLASTP analysis, vB_KleM-RaK2 protein 041 (888 aa) exhibits 99% aa sequence identity (E-value of 0.0) to the tail sheath protein of *Klebsiella* phage K64-1 [30]. It also shares similarities with the tail sheath proteins of various bacteriophages (Figure 1) and bacterial homologues.

**Figure 2:**
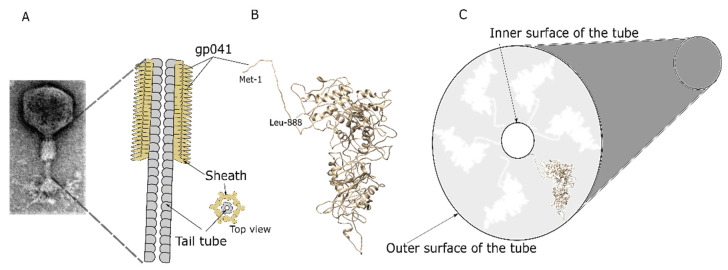
Schematic representation of structures formed by protein 041. TEM image of bacteriophage vB_KleM-RaK2 and schematic view of its contracted sheath (**A**); a model of 041 protein generated by the PS^2^ server (**B**); the N- and C-terminal amino acids are indicated; the possible tube scheme based on the known structures of the tail sheaths (**C**).

The HHpred analysis revealed that only the C-terminus of protein 041 demonstrated the reliable identity of a number of already known structures: the fragment of aa from 589 to 888 of protein 041 had the best hit to the tail sheath protein (gp18) of *Escherichia* virus T4 (3J2M_V; identity, 24%; probability, 100%; E-value, 2.1 × 10^−33^). Studies of the three-dimensional structure showed that tail sheaths of *Myoviridae* possessed a sixfold rotational symmetry and that both ends of the tail sheath proteins were located inside the tube [35,36]. A 3D model of protein 041 revealed that both the N- and C-ends were oriented in the same direction, and by analogy with other tail sheath models, the N- and C-ends should be inside of the formed tube (Figure 2).

For gene expression and analysis, *orf041* was cloned into the expression vectors, and protein was synthesized in *E. coli* BL21 (DE3) cells. The recombinant protein 041 was obtained in both soluble and insoluble protein fractions (Supplementary Figure S1). According the TEM analysis, the tubular structures were observed in the soluble protein fraction. The length of these structures varied from 10 nm to 750 nm, and the diameter (41 ± 5 nm) corresponded to the width of a contracted tail sheath (41 nm) of the phage vB_KleM-RaK2. Sucrose density gradient ultracentrifugation made it possible to obtain almost pure nanostructures.

Since we planned to construct chimeric proteins on the basis of the 041 protein, some additional space was needed to arrange the functional proteins inside the tube. Therefore, protein 041 was truncated by 100, 150, and 200 amino acids at the C-terminus, cloned to the expression vector, and synthesized in *E. coli* cells. The TEM analysis of soluble protein fraction revealed that the loss of 100 to 200 amino acids still allowed protein 041 to form nanotubes (Supplementary Figure S2). These results were in accordance with a previous observation that deletions of residues from the C-terminus of the tail sheath protein (gp18) from phage T4 only slightly reduced its polymerization ability [37,38].

### 3.2. Chimeric Protein Construction

**Figure 3:**
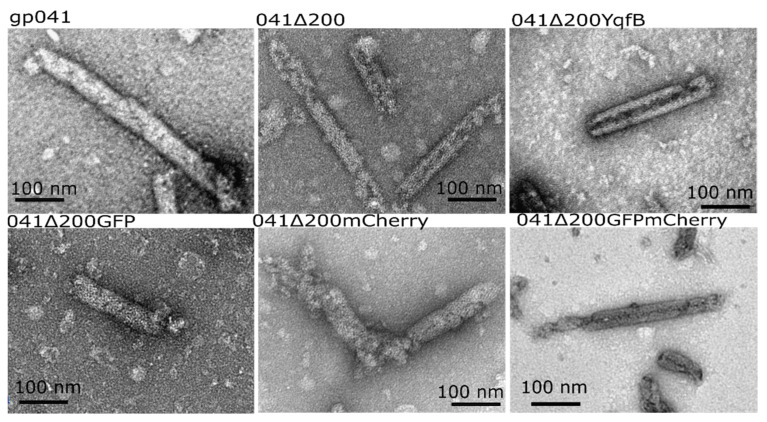
Electron micrographs of nanostructures formed by recombinant protein 041 and its modified variants.

For further studies, the 200 amino-acid-truncated protein 041 (041Δ200) was chosen. To construct the chimeric nanotubes, two fluorescent proteins (GFP and mCherry) and amidohydrolase YqfB were fused to the C-terminus of 041Δ200 (The schematic model of constructed genes are represented in Supplementary Figure S3). All three chimeric proteins were successfully produced in *E. coli* cells and formed self-assembled nanotubes. Two fluorescent proteins, 041Δ200GFP and 041Δ200mCherry, were coexpressed in *E. coli* cells and formed nanotubes as well (Figure 3). The length of the formed chimeric nanotubes was similar to the length of the wild-type nanotubes, though diameter differed (Table 3, Supplementary Figure S4). A total 93 ± 3% of chimeric proteins was assembled in the nanotubes (Supplementary Table S2).

**Figure 4:**
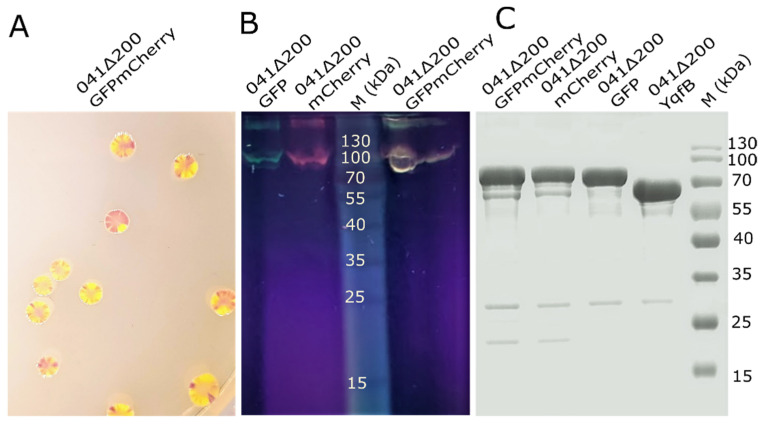
Analysis of chimeric 041Δ200 expression. (**A**) The individual colonies of *E. coli* BL21 (DE3) coexpressing 041Δ200GFP and 041Δ200mCherry; (**B**) purified chimeric 041Δ200GFP and 041Δ200mCherry proteins in polyacrylamide gel under UV light; (**C**) PAGE analysis of the purified 041Δ200GFP, 041Δ200mCherry, and 041Δ200YqfB proteins.

041Δ200GFP and 041Δ200mCherry-expressing bacterial colonies and liquid culture cells, as well as the purified nanotubes, were fluorescent, and the cells coexpressing chimeric 041Δ200GFP and 041Δ200mCherry proteins formed fluorescent bicolored colonies (Figure 4A). The fluorescence intensities of the two proteins differed in different colonies.

For the purification of chimeric nanotubes, sucrose density gradient ultrafiltration was effective (Figure 4C). When purified fluorescent nanotubes were loaded onto SDS-PAGE without denaturation and the gel was exposed to the UV-light (Figure 4B), the fluorescence band matched the band of purified chimeric proteins. Some of the nanostructures were not denatured and stuck on the gel.

### 3.3. Analysis of 041Δ200GFP and 041Δ200mCherry Nanotubes

**Figure 5:**
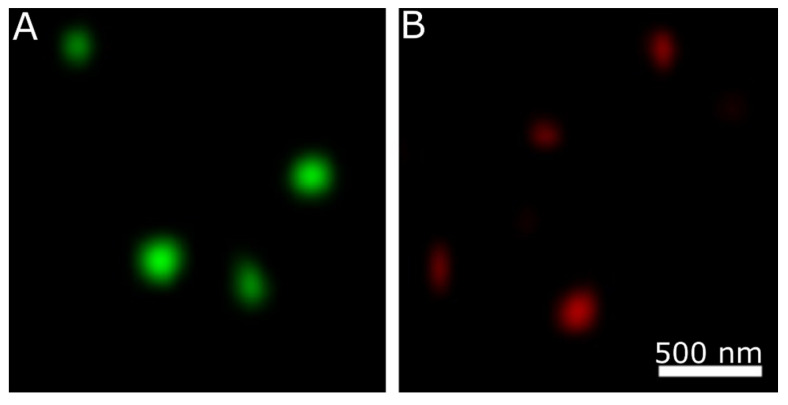
Fluorescence analysis of nanotubes. (**A**) Nanotube formed by 041Δ200GFP; (**B**) nanotube formed by 041Δ200mCherry.

To make sure that the fluorescence was triggered by the tubes, the samples of chimeric proteins were analyzed by confocal microscopy. Analysis revealed that both proteins formed fluorescent structures (Figure 5). The size of individual structures corresponds the nanotubes’ size observed by the TEM.

**Figure 6:**
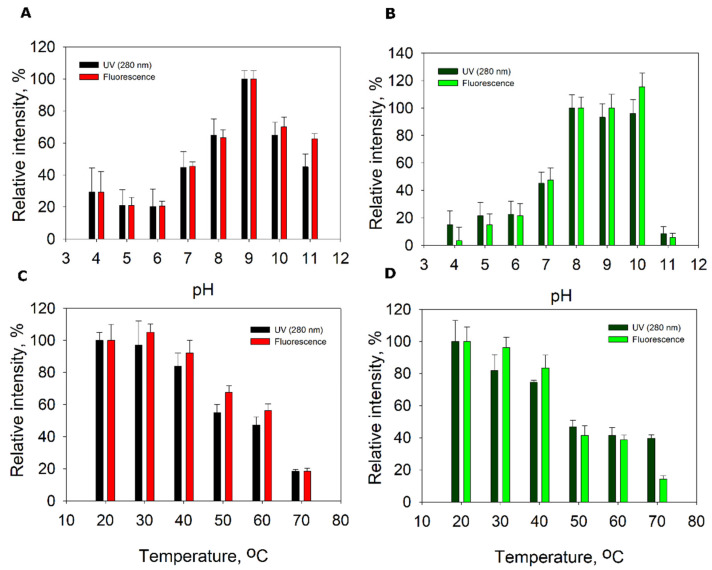
pH (**A**,**B**) and temperature (**C**,**D**) stability of hybrid 041Δ200GFP (**B**,**D**) and 041Δ200mCherry (**A**,**C**) nanotubes. The integrated areas of 10 min peak of absorbance at 280 nm and fluorescence at 610 nm (mCherry) or 506 nm (GFP) are shown.

We investigated the stability of fluorescent nanotubes by incubating the purified nanotubes at different temperatures or pH for 1 h and by analysis using size exclusion chromatography (SEC). It turned out that the tubes were sensitive to elevated temperature and acidic pH (Figure 6). Under optimal conditions, the purified nanotubes formed one fluorescence peak in the SEC analysis, with a retention time of 10 min. If the sample was exposed to unfavorable conditions, additional peaks with lower molecular weight were formed, indicating the decomposition of nanotubes. If the aggregation of nanotubes occurred in the samples, the formed precipitates were removed by centrifugation prior to SEC and were not visible in the analysis.

It turned out that at pH > 10 the nanotubes started to decompose. At lower pH (4–6) and elevated temperature, besides decomposition, a precipitate was formed in the samples, indicating an aggregation of the nanotubes.

### 3.4. Analysis of 041Δ200GFPmCherry Hybrid Nanotubes

**Figure 7:**
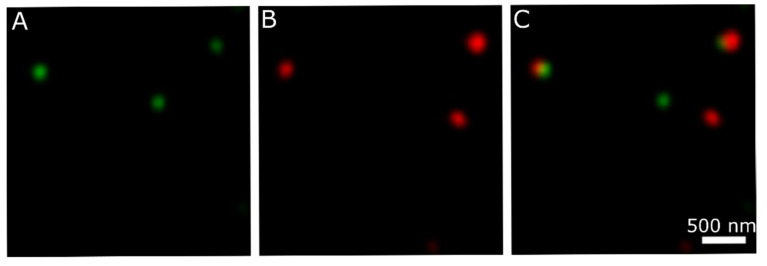
Fluorescence analysis of hybrid nanotube 041Δ200GFPmCherry. (**A**) Recorded GFP fluorescence signal; (**B**) recorded mCherry signal; (**C**) overlay of (**A**) and (**B**).

When the hybrid nanotubes containing two different fluorescent proteins were investigated, it turned out that both GFP and mCherry were fluorescent in one observed spot (Figure 7). This might indicate that both fluorescent proteins assembled in the same nanotube.

**Figure 8:**
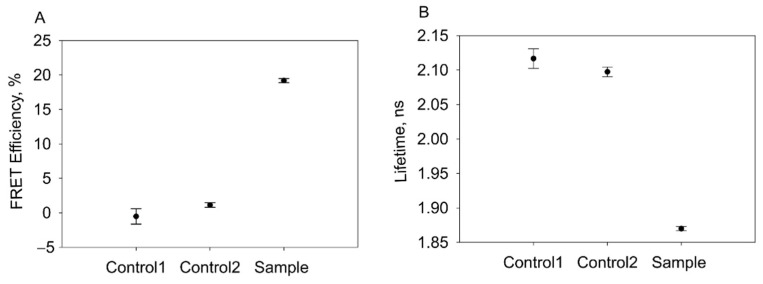
(**A**) FRET efficiency in nanotubes and (**B**) change in mean fluorescence lifetime in nanotubes. Control 1–041Δ200GFP, Control 2–041Δ200GFP mixed with 041Δ200mCherry (1:1), sample–041Δ200GFPmCherry nanotubes.

To show a proximity of both fluorophores, the FRET interaction between two fluorescent proteins within nanotubes was probed selectively, exciting GFP at 488 nm and observing its lifetime (Figure 8). The efficiency of FRET was calculated assuming a lifetime of 2.1 ns for the ‘unquenched’ donor (based on an acceptor-free control). When both fluorophores were in the same tube, the efficiency of FRET increased, and the average lifetime of GFP decreased, proving that energy transfer occurs between fluorophores. A sample in which both proteins resided on different assemblages (041Δ200GFP mixed with 041Δ200mCherry) showed only a small decrease in fluorescence lifetime and a low increase in FRET efficiency (Figure 8).

### 3.5. Analysis of 041Δ200YqfB Nanotubes

The purified 041Δ200YqfB nanotubes hydrolyzed *N*^4^-acetyl-2′-deoxycytidine. The specific activity of tube-fused YqfB was 33 U/mg of protein. Although the specific activity was three-fold lower than that of the wild-type YqfB, the turnover number of 041Δ200YqfB exceeded the turnover number of free YqfB by more than twice. Hence, both enzymes showed a similar catalytic efficiency (Table 4).

**Figure 9:**
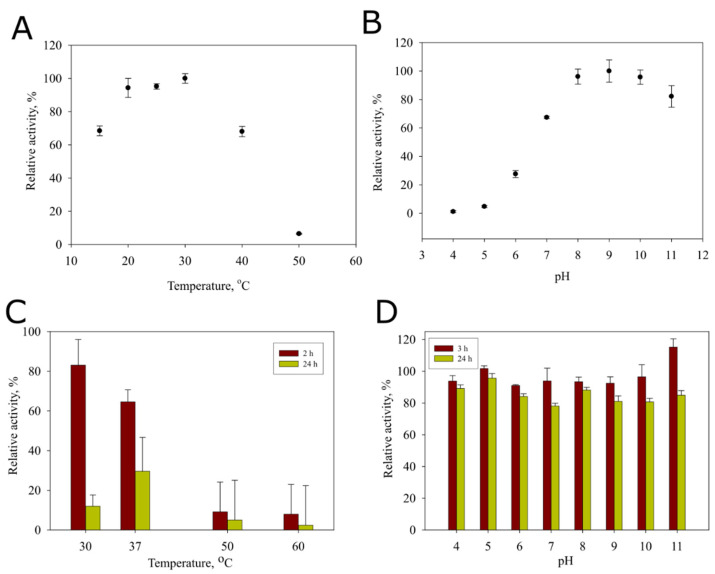
Effect of temperature and pH on activity and stability of 041Δ200YqfB. (**A**,**B**)—activity of 041Δ200YqfB towards *N*^4^-acetyl-2′-deoxycytidine at different temperatures and pH, respectively. (**C**,**D**)—a residual activity of the enzyme incubated at different temperatures or pH for 2–24 h, respectively.

We investigated the effect of temperature on purified tube-fused YqfB and found an increase in amidohydrolase activity from 20 to 30 °C, followed by a decline in activity at 40–50 °C (Figure 9A). Thermostability studies revealed that the enzyme lost activity at 50–60 °C after incubation for 2 h and was completely inactivated at 30 °C after incubation for 24 h (Figure 9C). The tube-fused YqfB had a pH optimum at 9.0 (Figure 9B) and retained more than 80% of its activity at pH values ranging from 4.0 to 11.0 within 3 and 24 h at 10 °C (Figure 9D).

## 4. Discussion

In this study, we examined the possibility of generating functionalized nanoparticles from the self-assembling bacteriophage protein. We chose a protein-fusion strategy for function establishment and the bacteriophage vB_KleM-RaK2 tail sheath protein 041 as a scaffold. The truncation of protein 041 at C-terminus by 200 amino acids (041Δ200) did not limit the ability of the shorter protein to self-assemble into nanotubes. Two distinct types of proteins were selected for the fusion: fluorescent proteins (GFP and mCherry) and amidohydrolase (YqfB). GFP and mCherry were directly fused to the truncated 041 tail sheath protein (041Δ200), and YqfB was fused with flexible glycine-rich linker. The chosen proteins were successfully expressed as 041 fusions and assembled into the protein nanotubes. Up to 4 mg of chimeric proteins from 50 mL of cultures was purified by simple sucrose density gradient centrifugation. The fluorescent proteins in the nanostructure maintained their characteristic absorbance and emission profiles, and the YqfB maintained the amidohydrolase activity. This indicates that the fused proteins were folded into their fully functional forms. The modeling of 041 protein shows that both the N- and C-termini are directed toward the inner space of the formed tube. Thus, the target protein was likely located inside of the tube. To the best of our knowledge, the obtained nanostructures are the first examples of the functionalization of protein nanotubes formed by hybrid tail sheath proteins.

The diameter of the inner space of the native 041 nanotubes is about 6 nm. We truncated the C-terminus of the 041 protein, aiming to increase the internal diameter of the protein nanotube and used this enlarged space for the arrangement of the functional proteins. The fused proteins did not affect the 041 protein’s ability to self-assemble into the tubes but slightly changed the morphology of the formed structures. Hence, all the fused proteins expanded the tube diameter. The most prominent changes were observed in the case of YqfB, which enlarged the tube diameter by almost 10 nm (a two-fold internal diameter increase) and formed a clearly visible central channel after a negative staining. The three-dimensional structures of GFP and mCherry are similar; still, the geometry of proteins differs, and so do the amino acids located on the surface of those proteins. The GFP (PDB: 2Y0G) should occupy 28.1 nm^3^, mCherry (PDB: 2H5Q)–22.6 nm^3^ and YqfB–12.8 nm^3^ [39]. Judging by the three-dimensional model, protein YqfB was the smallest one used in this study; however, it changed the nanotube’s structure the most. The alteration of morphology of nanotubes may be determined not only by the size but also by the shape or surface charge of the attached proteins. To answer these questions, further studies are required to elucidate the 3D structures of the hybrid proteins and the protein nanotubes themselves.

In addition, it was shown that two fluorescent proteins, 041Δ200GFP and 041Δ200mCherry, could be produced in one bacterial cell. The resulting bicolored bacterial colonies had segmented coloration, indicating that the ratio of recombinant proteins produced in each cell was different. This may indicate that 041Δ200GFP and 041Δ200mCherry proteins assemble into nanotubes randomly. To elucidate whether hybrid nanotubes were formed, FRET studies were carried out. The analysis of the collected data indicates a close distance between donor and acceptor chromophores, as well as assembly of 041Δ200GFP and 041Δ200mCherry into one nanotube. However, the assembly process cannot be fully controlled under the conditions we studied, and additional studies are required to optimize this process.

Self-assembling biocatalytic systems are much desired both for organic syntheses and prodrug activation. A detailed analysis of features of the tail sheath protein-fused amidohydrolase YqfB showed that the specific activity and kinetic constants of the enzyme did not change drastically. The pH optimum remained similar (pH 9 and pH 8 for 041Δ200YqfB (Figure 9) and wild-type YqfB [39], respectively), as did the catalytic efficiency. Some changes were observed regarding the temperature optimum (30 °C and 15 °C for 041Δ200YqfB and YqfB, respectively) and thermostability (10% and 40% of the remaining activity of 041Δ200YqfB and YqfB after incubation at 50 °C for 24 h, respectively) (Supplementary Figure S5). Other studies demonstrate that the immobilization of enzymes on viral particles is an attractive strategy for the development of versatile nanomaterials. Immobilization of some enzymes, for example, Lipase B [13] on potato virus X, or encapsulation of aspartate peptidase E [40] in Qβ, reduced the activity compared to the free enzymes. In some cases, the activity lowered slightly, such as in the case of cytosine deaminase encapsulated in SV40 [41], carbonic anhydrase fused to potato virus Y [14], or luciferase in Qβ [40]. In some cases, encapsulated enzymes seem to outperform the non-encapsulated ones, such as Lipase B in cowpea chlorotic mottle virus [42] or alcohol dehydrogenase D in bacteriophage P22 [43]. Besides, no increase in enzyme activity was observed with increasing enzyme content in the virus particle. In those cases, the inner space of the virus capsid is limited in entering a sufficient amount of substrate; thus, the concentration of encapsulated enzymes is higher than the substrate concentration inside the capsid. Therefore, the conversion rate of the substrate molecules does not change when there are more enzymes in the capsid, thus decreasing the overall enzyme conversion rate. In the case of 041Δ200YqfB, the catalytic rate was twice as high as that of the free YqfB. This could be due to the better positioning of the enzyme in the tube, or the crowding effect may be responsible for the increase in turnover number, as in the case of Lipase B and alcohol dehydrogenase D [42,43]. The higher K_m_ of nanotube-fused YqfB may be caused by the hindered access of the substrate to the active site.

## 5. Conclusions

Our study demonstrated that the bacteriophage tail sheath protein can be successfully used for generation of functionally active nanomaterials. A formation of protein nanotubes harboring two different hybrid proteins, as well as a formation of catalytically active assemblages, opens a possibility to create nanostructures for biocatalytic cascades. In addition, the developed self-assembling nanoparticles could be further optimized for enzyme or drug transportation into eukaryotic cells. Keeping in mind a different morphology of such particles compared to spherical ones, a different specificity and efficiency of the target recognition might be expected.

## Figures and Tables

**Table 1:** Primers used for amplification of genes. The restriction endonuclease recognition sites are underlined.

Primer	Sequence 5′-3′	Resulting Plasmid
041XhoR	gtgctcgagagtattttcaatac	pET21_041
041NcoF	tataccatggcagatttaatcag	
041Δ200HindR	gccaagctttctagtactcaggc	pET28_041Δ200
mcheryHindF	cgtgtaaagcttgttagtaaaggagaaga	pET21_041Δ200mCherry
mcheryXhoR	tagttctcgagttatgcggtaccaga	
gfpHindF	agaaggaaagcttcatatgagtaaagga	pET28_041Δ200GFP
gfpXhoR	gtgctcgagtgatttgtatag	
YqfbbukF	atgcagccaaacgacatcac	pET21_041Δ200YqfB
YqfbbukR	ttaaagacatttaaattcaatcacata	
GS_F	agcttggaggaggaggaagtggaggaggaggaagtcctagggtcgactacgtac	pET21_041Δ200GS
GS_R	tcgagtacgtagtcgaccctaggacttcctcctcctccacttcctcctcca	

**Table 2:** Fluorescence parameters used for chimeric protein analysis.

Protein	Excititation (nm)	Emission (nm)
041Δ200GFP	488	493–582
041Δ200mCherry	587	600–700

**Table 3:** Characterization of the recombinant nanotubes.

Construct	Length, nm	Diameter, nm
041	319.7 ± 76.1	41.1 ± 4.9
041Δ200	311.7 ± 87.7	43.4 ± 4.2
041Δ200GFP	276.0 ± 68.2	43.9 ± 4.1
041Δ200mCherry	237.3 ± 76.4	49.2 ± 3.4
041Δ200GFPmCherry	283.8 ± 86.2	46.3 ± 3.4
041Δ200YqfB	231.0 ± 106	51.5 ± 9.4 (internal diameter 13 nm)

**Table 4:** Comparison of catalytic parameters of free YqfB and tube-fused YqfB on *N*^4^-acetyl-2′-deoxycytidine.

Protein	Activity, U/mg	K_m_, M	k_cat_, s^−^^1^	k_cat_/K_m_, M^−^^1^s^−^^1^
041Δ200YqfB	33 ± 4.3	(7.6 ± 0.05) × 10^−4^	241 ± 2	3.2 × 10^5^
YqfB	101 ± 9.2	(4.9 ± 0.04) × 10^−4^	113 ± 1	2.3 × 10^5^

## Data Availability

Data are contained within the article or Supplementary Materials.

## Supplementary Materials

The following are available online at https://www.mdpi.com/article/10.3390/nano11113031/s1, Figure S1: SDS PAGE analysis of protein 041 synthesis in *E. coli* BL21 (DE3) cells. Table S1: Primers used for cloning of truncated 041 gene variants. Figure S2: TEM analysis of nanotubes of recombinant protein 041 and its truncated variants. Figure S3: Schematic figure of constructed chimeric genes. Figure S4: TEM analysis of chimeric nanotubes. Table S2: Analysis of assembly of recombinant chimeric proteins. Figure S5: Thermostability of 041Δ200YqfB and YqfB after 2 and 24 h of incubation.
