# Supplementary material for: Functionalized Protein Nanotubes Based on the Bacteriophage vB_KleM-RaK2 Tail Sheath Protein
Source: Nanomaterials (Basel). 2021 Nov 12;11(11):3031. doi: 10.3390/nano11113031 (PMC8618960; doi:10.3390/nano11113031)
Supplement: Supplementary file 1 [file nanomaterials-11-03031-s001.zip › nanomaterials-1450073-supplementary.pdf]

Supplementary materials

Functionalized Protein Nanotubes Based on the Bacteriophage vB_KleM-RaK2 Tail Sheath Protein

Greta Labutytė [1], Simona Povilonienė [1], Eugenijus Šimoliūnas [1], Dovydas Gabrielaitis [1,2], Martynas Skapas [3],
Algirdas Noreika [1], Rolandas Meškys [1,*] and Vida Časaitė [1,*]

[1] Department of Molecular Microbiology and Biotechnology, Institute of Biochemistry, Life Sciences Center, Vilnius University, Sauletekio av. 7, LT-10257 Vilnius, Lithuania; greta.labutyte@gmc.stud.vu.lt (G.L.); simona.poviloniene@bchi.vu.lt (S.P.); eugenijus.simoliunas@bchi.vu.lt (E.Š.); dovydas.gabrielaitis@gmc.vu.lt (D.G.); algirdas.noreika@gmc.vu.lt (A.N.)

[2] Department of Neurobiology and Biophysics, Institute of Biosciences, Life Sciences Center, Vilnius University, Sauletekio av. 7, LT-10257 Vilnius, Lithuania

[3] Center for Physical Sciences and Technology, Saulėtekio av. 3, LT-10257 Vilnius, Lithuania; martynas.skapas@ftmc.lt

* Correspondence: rolandas.meskys@bchi.vu.lt (R.M.); vida.casaite@bchi.vu.lt (V.Č.)

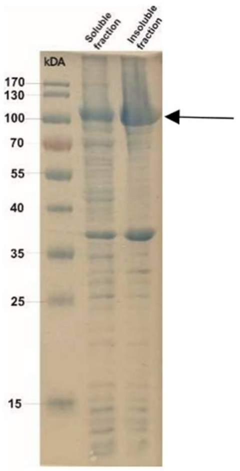

Figure S1. SDS-PAGE analysis of protein 041 synthesis in *E. coli* BL21 (DE3) cells. Soluble and insolublecell-free extracts are indicated; arrow shows the 041 protein corresponding band.

Table S1. Primers used for cloning of the truncated 041 gene variants. The restriction endonucleaserecognition sites are underlined.

Primer	Sequence 5'-3'	Resulting plasmid
041NheF	5′ GGAGGAGCTAGCATGGCAGATTTA 3′	
041Δ100HindR	5′GACATTAAGCTTTCATAGTGCAGAAGT 3′	pET28_041Δ100
041Δ150HindR	5′ATATTCAAGCTTTCAGTTAATAAAACC 3′	pET28_041Δ150
041Δ200HindR	5′ AGTACCAAGCTTTCAAGTACTCAGGCA 3′	pET28_041Δ200
041Δ250HindR	5′ TAATCGCGCAAGCTTTCAAGTAATAAT 3′	pET28_041Δ250

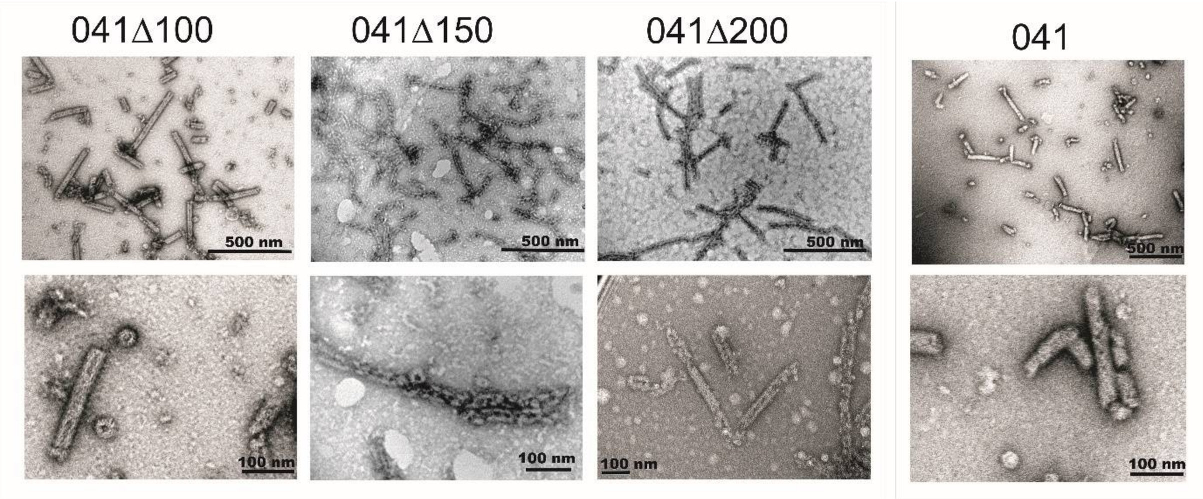

Figure S2. TEM analysis of nanotubes formed by the recombinant protein 041 and its truncated variants.

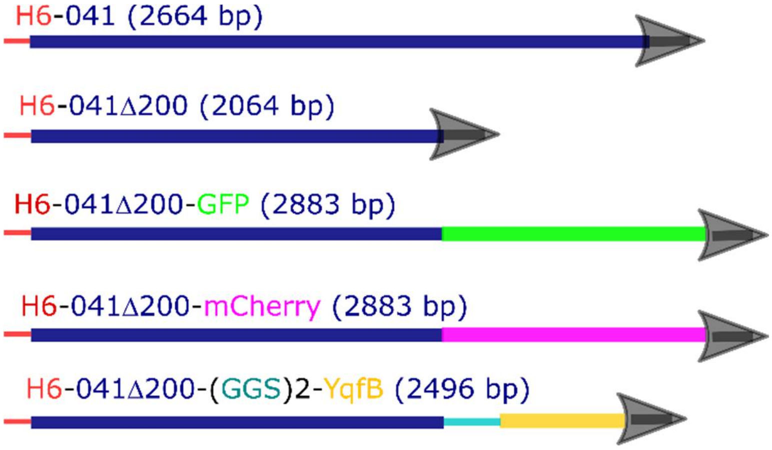

Figure S3. Schematic figure of constructed chimeric genes. H6 – 6-histidine tag; 041 – gp041; 041Δ200 – truncated gp041; (GGS) – linker; GFP, mCherry, YqfB – fused proteins. The arrow indicates the direction of gene translation. Gene sizes are indicated in parentheses.

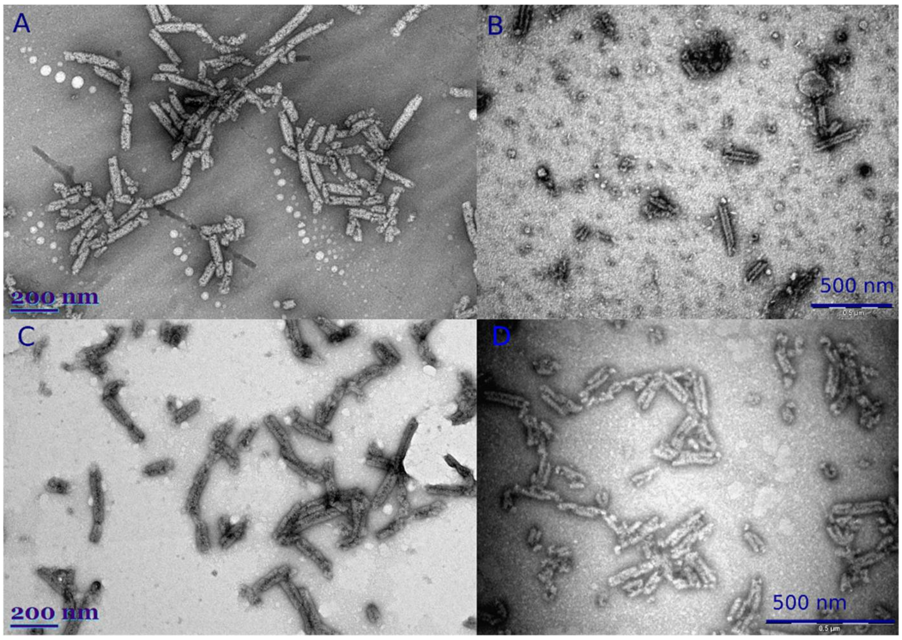

Figure S4. TEM analysis of chimeric nanotubes. A – 041Δ200GFP, B – 041Δ200YqfB, C – 041Δ200GFPmCherry, D – 041Δ200mCherry.

Table S2. Analysis of assembly of recombinant chimeric proteins.

**Protein**	**041ΔGFP***	**041ΔGFPmCherry***	**041ΔmCherry***	**041ΔmCherry****
Assembled in nanotubes (%)	96.7	91.3	90.9	87.4
Unassembled*** (%)	3.3	7.3 (mCherry)<br>1.5 (GFP)	9.1	9.6

*Cell-free protein extracts were analyzed by HPLC, obtained peaks of absorbance at 280 nm (corresponding the fluorescent peaks at 610 nm (mCherry) and 506 nm (GFP)) were integrated and used for evaluation.
**Purified nanotubes (pellets after ultracentrifugation) and unassembled protein (fluorescent supernatant) were analyzed by the SDS-PAGE and evaluated by the GelAnalyzer 19.1 (www.gelanalyzer.com) program. 500, 250, 125 ng of Bovine serum albumin was used for calibration curve.
***In the HPLC analysis the unassembled proteins were eluted in the form of hexamers.

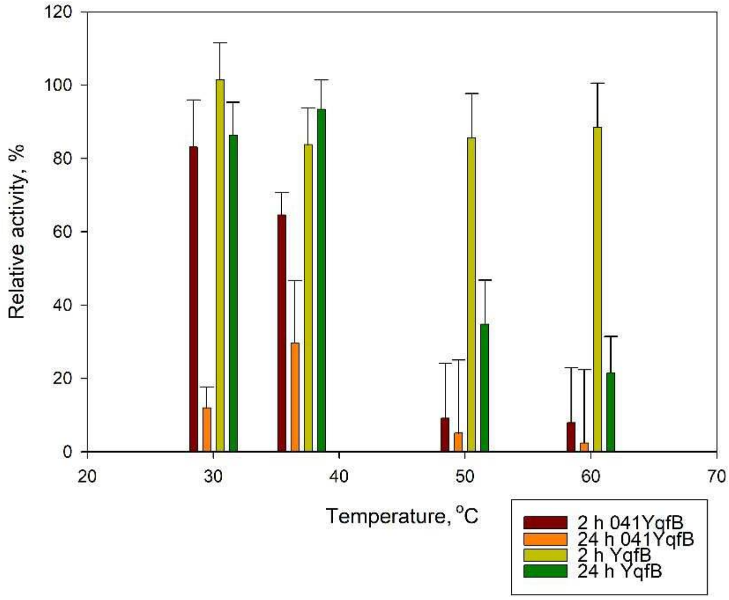

Figure S5. Thermostability of 041Δ200YqfB and YqfB. Activity of YqfB was analysed after 2 and 24 h of incubation.
